# Morphological Variation in Leaf Dissection of *Rheum palmatum* Complex (Polygonaceae)

**DOI:** 10.1371/journal.pone.0110760

**Published:** 2014-10-28

**Authors:** Xu-Mei Wang, Xiao-Qi Hou, Yu-Qu Zhang, Yan Li

**Affiliations:** 1 School of Pharmacy, Xi'an Jiaotong University, Xi'an, China; 2 Guiyang Museum of Medical Resources, Guiyang Botanical Garden of Medicinal Plants, Guiyang, China; 3 College of Life Sciences, Shaanxi Normal University, Xi'an, China; The National Orchid Conservation Center of China; The Orchid Conservation & Research Center of Shenzhen, China

## Abstract

**Aims:**

*Rheum palmatum* complex comprises all taxa within section *Palmata* in the genus *Rheum*, including *R. officinale*, *R. palmatum*, *R. tanguticum*, *R. tanguticum* var. *liupanshanense* and *R. laciniatum*. The identification of the taxa in section *Palmata* is based primarily on the degree of leaf blade dissection and the shape of the lobes; however, difficulties in species identification may arise from their significant variation. The aim of this study is to analyze the patterns of variation in leaf blade characteristics within and among populations through population-based sampling covering the entire distribution range of *R*. *palmatum* complex.

**Methods:**

Samples were taken from 2340 leaves from 780 individuals and 44 populations representing the four species, and the degree of leaf blade dissection and the shape of the lobe were measured to yield a set of quantitative data. Furthermore, those data were statistically analyzed.

**Important Findings:**

The statistical analysis showed that the degree of leaf blade dissection is continuous from lobed to parted, and the shape of the lobe is also continuous from broadly triangular to lanceolate both within and between populations. We suggested that taxa in section *Palmata* should be considered as one species. Based on the research on the *R. palmatum* complex, we considered that the quantitative characteristics were greatly influenced by the environment. Therefore, it is not reliable to delimitate the species according to the continuously quantitative vegetative characteristics.

## Introduction

Species are fundamental units of systematic, ecological and evolutionary studies, and species delimitation has long been the focus of biologists' attention. The discovery and description of species is a major endeavor in the field of systematics. However, most species continue to be circumscribed based on morphological comparisons of museum specimens [1]. Published descriptions of new plant and animal species almost always include a herbarium specimen designated as a holotype and a list of diagnostic morphological features [1]. It may be insufficient to delimit species merely based on herbarium specimens because such specimens usually represent discontinuous sampling and only a handful of individuals from a limited number of localities or populations. Discontinuous sampling may fail to reveal the total geographic-morphological variations that occur within species, and such ignored variations may represent important characteristics that demonstrate the continuity within/among populations. Discontinuous sampling may also lead to the publication of many microspecies within a genus, and these species are often later considered as a species complex. Therefore, the delimitation of species and infraspecific taxa should be based on the study of natural populations [2].

For some genera of Chinese flora, microspecies were published by several authors based on the limited specimens collected from various localities by different collectors. Chinese botanists have devoted attention to some species complexes in recent decades [3]–[19], but only a small number of taxa have been investigated. *Rheum palmatum* complex comprises all taxa (i.e., *R. officinale* Baill., *R. palmatum* Linn., *R. tanguticum* Maxim. ex Balf., *R*. *tanguticum* var. *liupanshanense* C. Y. Cheng et T. C. Kao and *R. laciniatum* Prain) within *Rheum* Sect. *Palmata*. These taxa differ from other members of genus *Rheum* in the possession of palmately lobed leaf blades [20], and they are monophyletic [21]. The key characteristics within the species complex are the degree of leaf blade dissection and the lobe shape [20]. The blades of *R. officinale* and *R. palmatum* are lobed; the lobes of *R. officinale* are broadly triangular, and those of *R. palmatum* are narrowly triangular. The blades of *R. tanguticum* and *R. laciniatum* are parted, and the lobes are narrow and triangular-lanceolate. The difference between *R. tanguticum* and *R. laciniatum* is that the lobelets of the former are narrowly lanceolate, and those of the latter are linear. However, the transitional morphologies between species are often observed in both the herbarium specimens and the field individuals. Previous DNA-based studies have drawn conflicting conclusions, and the interspecific relationships remain to be resolved. Using AFLP (amplified fragment length polymorphism) markers, Suo et al. [22] concluded that *R*. *officinale* and *R*. *palmatum* are mutually sisters and are together sisters with *R*. *tanguticum* based on samples from Sichuan and Gansu provinces. Using DNA sequences from the chloroplast gene *mat*K, Yang et al. [23] reported that *R*. *officinale* and *R*. *tanguticum* are mutually sisters and are together sisters with *R*. *palmatum* based on samples from Qinghai, Sichuan, and Gansu provinces. Based on chloroplast DNA *trn*L*-*F sequences from samples from Chongqing and Qinghai provinces, Wang et al. [21] showed that the individuals of *R*. *palmatum*, *R*. *officinale*, and *R*. *tanguticum* are nested one with one another on the phylogenetic tree. Based on the nuclear ITS (internal transcribed spacer) sequences from eight samples from Qinghai and Gansu provinces, Li et al. [24] indicated that the only sample of *R*. *officinale* in their study is sister with their seven samples of *R*. *palmatum* and *R*. *tanguticum*. According to our previous study, *R. palmatum* is the most widely distributed among the distribution of the species complex; the distribution of *R*. *tanguticum* overlaps with that of *R*. *palmatum* in northwestern distribution of the species complex, the distribution of *R*. *officinale* overlaps with that of *R*. *palmatum* in southeastern distribution of the species complex, and the distribution of *R*. *officinale* also overlaps with that of *R*. *tanguticum* [25]. *R. laciniatum* only inhabits the northern Sichuan province and embeds in the distribution areas of the other three species [20]. Based on their geographic distribution patterns and other evidence, we propose that these four species of Sect. *Palmata* might not be truly distinct species [25] and that this characterization might be attributable to the discontinuous sampling. The key characteristics (e.g., the degree of leaf blade dissection and the shape of the lobes) among the species in the complex are quantitative. Therefore, the main objective of the present study is to analyze the pattern of variation of these characteristics within and among populations through population-based sampling covering the entire distribution range of *R*. *palmatum* complex.

## Materials and Methods

### Ethics statement

According to regulations of the People's Republic of China on the protection of wild plants, permits are required only for the species included on the list of state-protected plant species at the time of collection. Sect. *Palmata* is not on the list of state-protected plant species [26] (Regulations of the People's Republic of China on the protection of wild plants, http://www.people.com.cn/item/faguiku/zrzyf/U1020.html). Thus, no specific permits were required for the described field studies, and no harm was caused to the plants and their habitats.

### Materials

**Figure 1:**
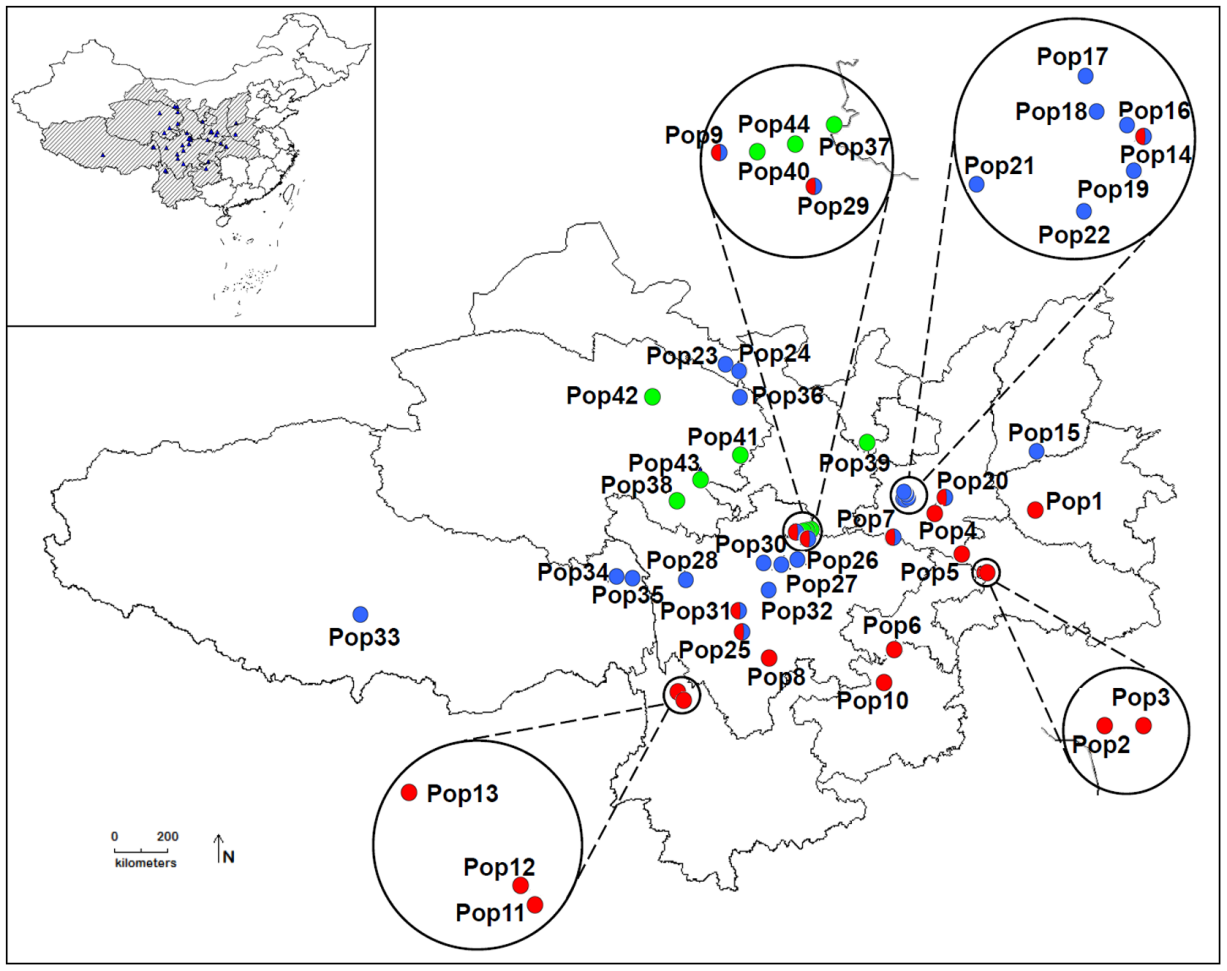
Geographic distribution of the 44 studied populations of *Rheum palmatum* complex.

Morphological analyses were conducted on 2340 leaf blades from 780 individuals from 44 populations in the field, representing all taxa within *R*. *palmatum* complex, i.e., *R. officinale*, *R. palmatum*, *R. tanguticum*, *R. tanguticum* var. *liupanshanense*, and *R. laciniatum*, from the entire distribution range of Sect. *Palmata* (Fig. 1, Table 1). Vouchers were deposited in the herbarium of Shaanxi Normal University (SNNU) (Xi'an, China).

**Table 1:** Studied population information of *R. palmatum* complex.

Population	Locality	Altitude (m)	Longitude (°N)	Latitude (°E)	No. of individuals
Pop1	Baotianman, Mt. Funiu, Neixiang County, Henan Province	1100	33.486300	111.916000	11
Pop2	Muyu, Mt. Shennongjia, Xingshan County, Hubei Province	2575	31.450700	110.187150	5
Pop3	Muyu, Mt. Shennongjia, Xingshan County, Hubei Province	2908	31.446000	110.268000	17
Pop4	Taibaimiao, Ningshan County, Shaanxi Province	1878	33.423433	108.530550	20
Pop5	Mt. Hualong, Baxian, Pingli County, Shaanxi Province	2919	32.023217	109.358320	19
Pop6	Daping, Mt. Jinfo, Nanchuan, Chongqing municipality	1412	28.973567	107.183720	19
Pop7	Yingshuiba, Guanba, Nanjiang County, Sichuan Pro vince	1809	32.594472	107.113000	19
Pop8	Hongxi Forest Farm, Meigu County, Sichuan Province	3623	28.670717	102.972350	16
Pop9	Sigou, Huanglong, Songpan County, Sichuan Province	2831	32.797633	103.581030	20
Pop10	Hailongtun, Gaoping, Zunyi County, Guizhou Province	1252	27.812767	106.818280	8
Pop11	Haba, Sanba, Xianggelila County, Yunnan Province	3995	27.386983	100.045900	16
Pop12	Haba, Sanba, Xianggelila County, Yunnan Province	3727	27.395683	100.037620	16
Pop13	Xiaozhongdian, Xianggelila County, Yunnan Province	3441	27.585306	99.847250	20
Pop14	Xiabansi, Mt. Taibai, Mei County, Shaanxi Province	2807	33.969600	107.794200	20
Pop15	Shuiwangping, Zhongchun, Qinshui County, Shanxi Province	1766	35.418883	111.954320	20
Pop16	Wengongmiao, Mt. Taibai, Mei County, Shaanxi Province	3423	33.978183	107.780170	20
Pop17	Doumugong, Mt. Taibai, Mei County, Shaanxi Province	2841	34.038100	107.714480	19
Pop18	Mingxingsi, Mt. Taibai, Mei County, Shaanxi Province	2859	33.996600	107.731630	20
Pop19	Nantianmen, Mt. Taibai, Zhouzhi County, Shaanxi Province	2652	33.921400	107.790000	20
Pop20	Mt. Guangtou, Fengyu, Chang'an County, Shaanxi Province	2578	33.870000	108.760000	20
Pop21	Haitanghe, Huangbaiyuan, Taibai County, Shaanxi Province	2400	33.899467	107.557980	20
Pop22	Longdonggou, Laoxiancheng, Zhouzhi County, Shaanxi Province	2658	33.866367	107.713600	18
Pop23	Heilingou, Mt. Qilian, Yongchang County, Gansu Province	2575	38.166667	101.433330	9
Pop24	Wangbalangyan, Mt. Qilian, Yongchang County, Gansu Province	3006	38.105040	101.862200	10
Pop25	Mt. Gongga, Luding coungty, Sichuan Province	3204	29.569611	101.983390	19
Pop26	Mt. Baoding, Baoding, Mao County, Sichuan Province	3102	31.932000	103.915400	14
Pop27	Naha, Sanlong, Mao County, Sichuan Province	2337	31.806583	103.53293	19
Pop28	Aji, Dagai, Xinlong County, Sichuan Province	3760	31.298767	100.05133	20
Pop29	Fenghe, Xiaohe, Songpan County, Sichuan Province	2749	32.601833	104.16242	20
Pop30	Miyaluo, Li County, Sichuan Province	3579	31.772139	102.75733	20
Pop31	Zhonggu, Yala, Kangding County, Sichuan Province	3692	30.246361	101.86389	20
Pop32	Mt. Balang, Wolong, Wenchuan County, Sichuan Province	3590	30.884139	102.96569	20
Pop33	Luoza, Rendui, Nanmulin County, Tibet Zang Autonomous Region	4498	30.131250	89.091700	20
Pop34	Tuoba, Changdu County, Tibet Zang Autonomous Region	4418	31.354733	97.690917	20
Pop35	Qingnidong, Jiangda County, Tibet Zang Autonomous Region	4000	31.376111	97.905556	18
Pop36	Qiaotan, Xianmi, Menyuan County, Qinghai Province	3148	37.192483	101.998900	18
Pop37	Qiujiaba, Tielou, Wen County, Gansu Province	3234	32.926556	104.287110	19
Pop38	Jimai, Dari County, Qinghai Province	3947	33.819183	99.711150	20
Pop39	Longwangmiaogou, Mt. Liupan, Jingyuan County, Ningxia Hui Autonomous Region	2224	35.666667	106.216670	13
Pop40	Yaogou, Huanglong, Songpan County, Sichuan Province	3597	32.797733	103.874480	19
Pop41	Maixiu Forest Farm, Zeku County, Qinghai Province	3349	35.314533	101.931200	20
Pop42	Mt. Guanjiao, Xinyuan, Tianjun County, Qinghai Province	3693	37.093500	98.856633	20
Pop43	Duocigou, Lajia, Jungong, Maqin County, Qinghai Province	3373	34.615917	100.566170	20
Pop44	Baishagou, Wanglang Reserve, Pingwu County, Sichuan Province	3193	32.873900	104.050170	19

### Measurement and analysis of leaf blade characteristics

**Figure 2:**
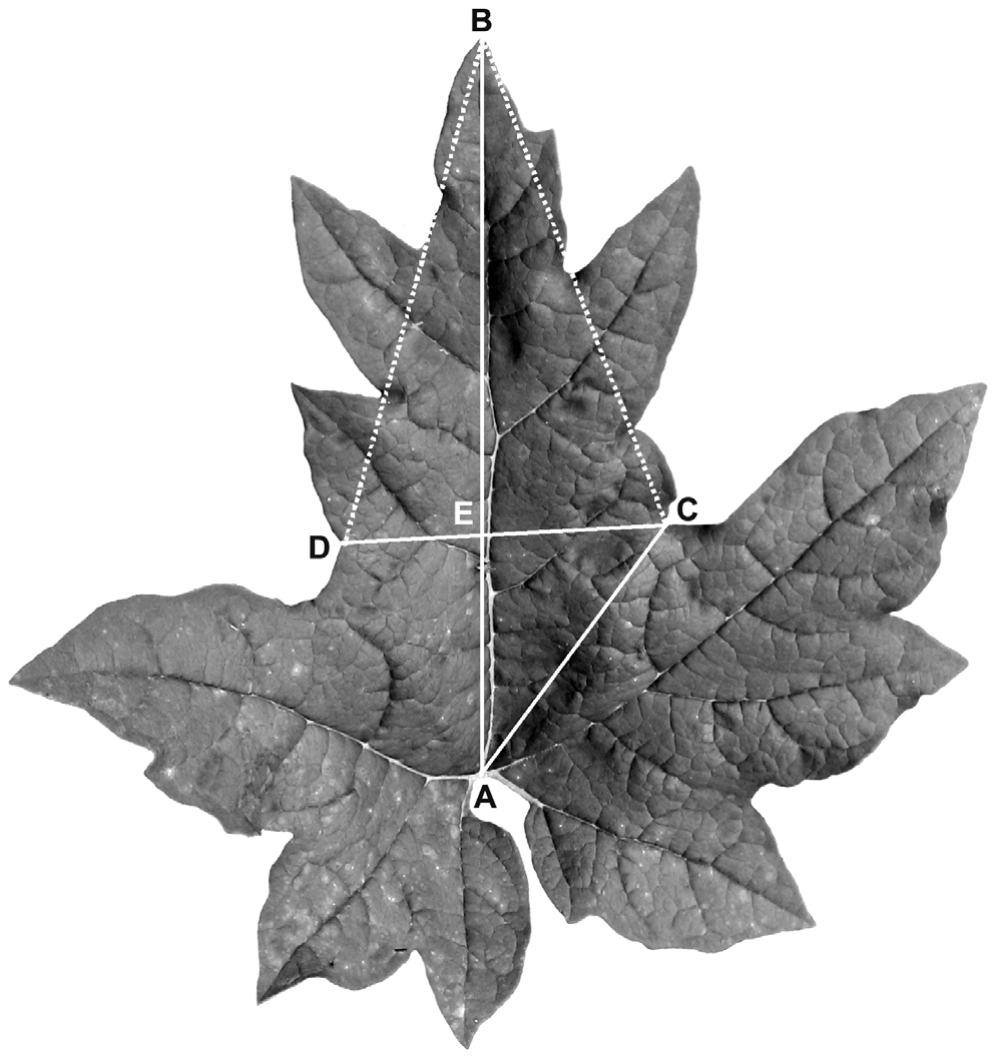
The measurement of a leaf blade of *Rheum palmatum* complex. A typical leaf form of No. 5 individual of Pop21.

The degree of leaf blade dissection and the shape of the lobes were measured. Because the two basal-most leaves of all investigated individuals are entire or waved, the third, fourth and fifth leaves from the bottom of the stem were measured in each individual. The mean value was obtained from three leaves for each individual. The leaf blades of the species complex are five-palmate lobed with five main veins, and the five lobes and veins were accordingly named the central, lateral and basal lobes and veins, respectively, and the clefts between the central and lateral and the lateral and basal lobes were called the lateral and basal clefts, respectively (Fig. 2).

To characterize the degree of leaf blade dissection and the shape of the lobes, the following parameters were measured on the leaf blade: AB, the length of the central vein, to represent the length of leaf blade; AC, from the connection point of the blade and petiole to the lowest point of the sinus between the central and lateral lobes, to represent the depth of the lateral cleft; CD, the distance between the two lowest points of the sinus of the two lateral clefts, to represent the width of the central lobe; and BE, the length of the central vein from the leaf blade apex (B) to CD, to represent the length of the central lobe (Fig. 2).

To intuitively indicate the changes in the leaf lobe width and the leaf cleft depth, the above lines (i.e., parameters) were linked to form two triangles, ABC and BCE. In triangle ABC, if the length of the central vein (AB) is regarded as a unit of length, then the values of AC/AB vary with the depth of the leaf cleft. A smaller AC/AB value indicates that the leaf cleft is deeper (i.e., parted), whereas a larger AC/AB value shows that the leaf cleft is shallower (i.e., lobed). Likewise, if BE, the height of triangle BCD, is regarded as a unit of length, then the values of CD/BE vary with the width of the leaf lobe. A smaller CD/BE value indicates that the leaf lobe is narrower (i.e., lanceolate), whereas a larger CD/BE value shows that the leaf lobe is wider (i.e., broadly triangular). Therefore, the analyses of inter-population and intra-population variations were conducted at the individual and population levels based on the values of AC/AB and CD/BE. Variation among the populations can be observed from the scatter diagram comparing the two indices of leaf lobes and from the histogram indicating the number of individuals in different ranges of leaf cleft indices. Variation within the populations can be obtained from the histogram indicating the range of variation of leaf cleft indices in different individuals.

Using the parameters measured in the field, the maximum and minimum values of AC/AB and CD/BE were obtained at the individual and population levels, and the difference between the maximum and minimum values for each parameter were then assessed. Because the scatter diagram showed that the AC/AB and CD/BE values among different populations were continuous within taxa of *R. palmatum* complex, the differences between the maximum and minimum values of AC/AB or CD/BE were divided into three equal intervals, and each interval corresponded theoretically to a morphological variation range of leaf blade. Thus, each morphological variation range of leaf blade was obtained. All measurements were obtained using a rule precise to 0.1 cm. The correlation analysis among the morphological parameters and the correlation analysis of these morphological parameters with environmental factors were performed with the statistical software SPSS 17.0 (Chicago, IL, USA).

## Results

### Leaf variations among populations

**Figure 3:**
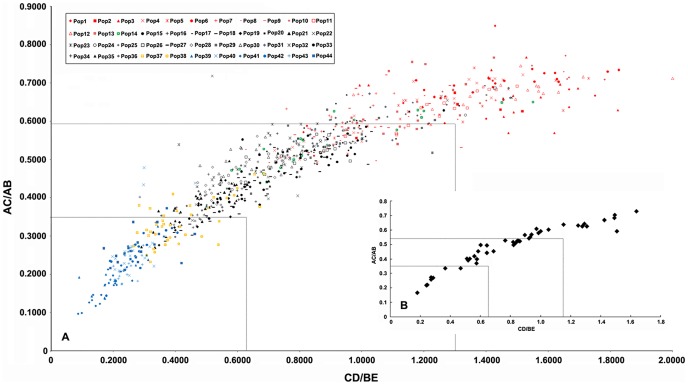
The scatter diagrams of AC/AB and CD/BE at the individual (A) and population (B) levels.

**Figure 4:**
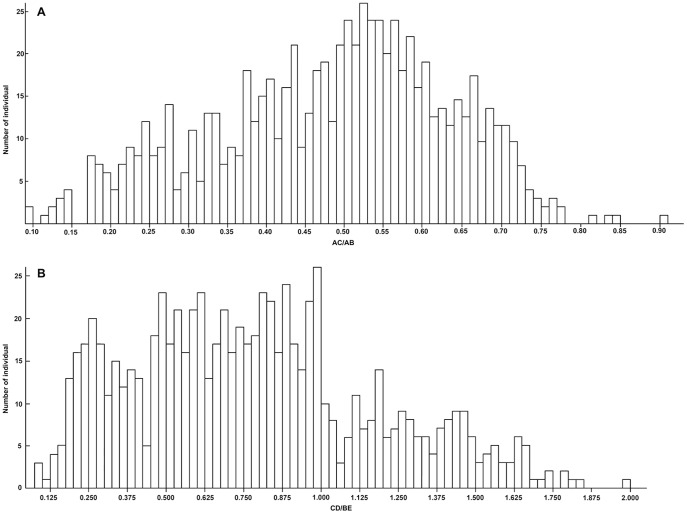
The numbers of individuals with different AC/AB (A) and CD/BE (B) values among the studied populations.

The scatter diagram showed that the values of both AC/AB and CD/BE were continuous at the individual (Fig. 3A) and population (Fig. 3B) levels and that neither distinct individuals nor populations could be clearly grouped. The two indices are significantly correlated (*r* = 0.949, *P*<0.01) (Table 2). If a leaf has a larger AC/AB value, then it must have a larger CD/BE value, indicating that the blade is lobed with broadly triangular lobes; in contrast, if a leaf has a smaller AC/AB value, then it must have a smaller CD/BE value, indicating that the blade is parted with lanceolate lobes. The number of individuals of different AC/AB values (Fig. 4A) showed a near-normal distribution, and the AC/AB values are not disconnected. In other words, some individuals in the *R. palmatum* complex possessed deeper leaf divisions, but most individuals were characterized by moderate leaf divisions. Despite also showing a continuous distribution, the pattern of CD/BE values (Fig. 4B) differed slightly from that of AC/AB values (Fig. 4A). Most individuals had smaller CD/BE values (<1.000).

**Table 2:** Correlation coefficients among the leaf morphological traits and the environmental factors of *R. palmatum* complex.

	CD/BE	AC/AB
AC/AB	0.949**	
Altitude	−0.331^*^	−0.322^*^
Latitude	−0.622**	−0.562**
Longitude	0.294	0.278

**, *Correlation is significant at 1% and 5% levels of probability, respectively.

### Leaf variations within populations

**Figure 5:**
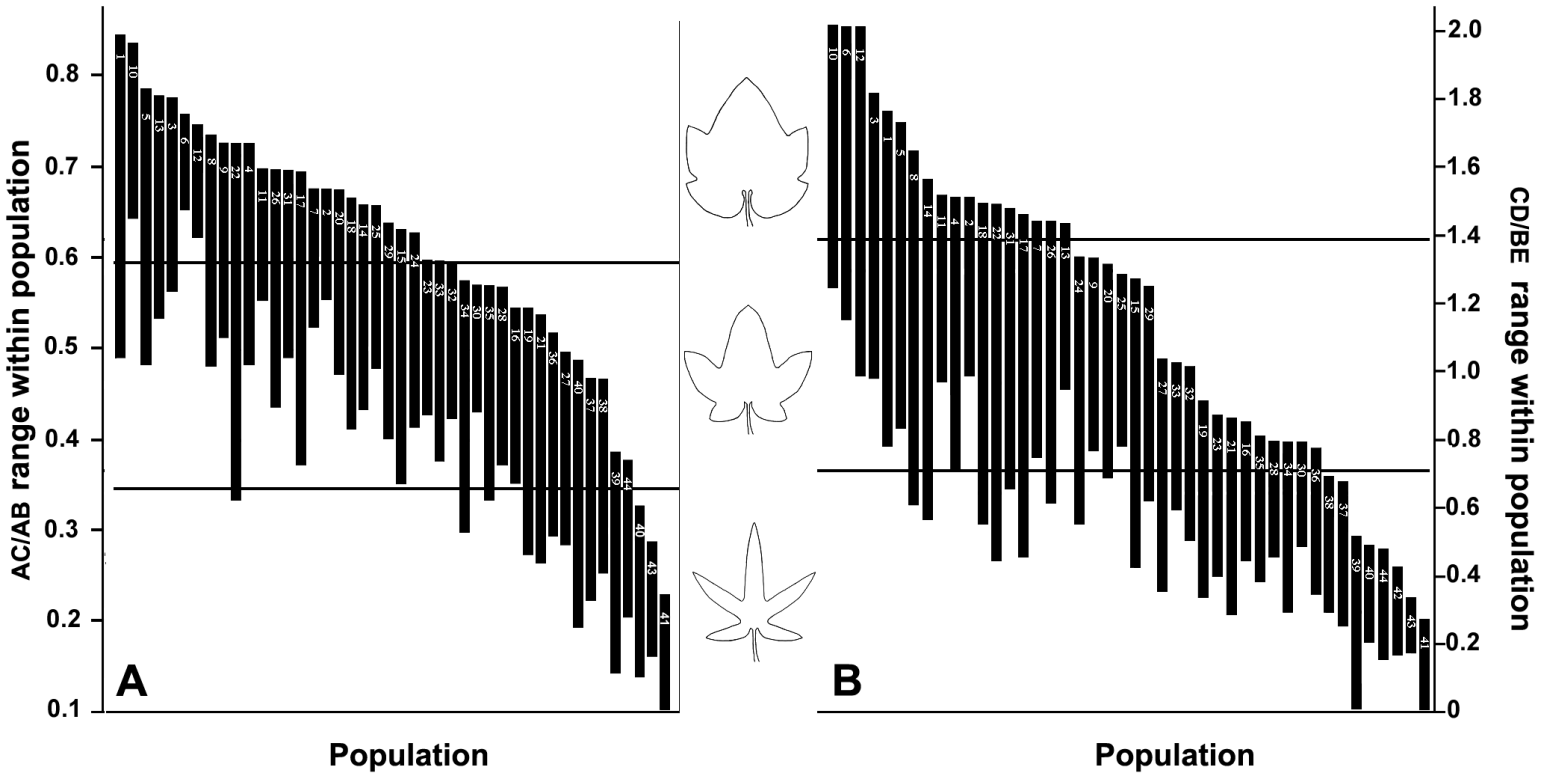
Range of variation of AC/AB (A) and CD/BE (B) values within the studied populations.

The AC/AB values ranged from 0.0970 to 0.8485 at the individual level in the *R. palmatum* complex, and the difference between the maximum and minimum values is 0.7515 (Tables 3, 4). As shown in the scatter diagram (Fig. 3), both AC/AB and CD/BE values were continuous in all taxa of the *R. palmatum* complex, which displayed three gradient-like ranges in the leaf morphology, and the median value of each range may represent a leaf blade type, i.e., lobed and broad triangular for *R*. *officinale*, lobed and triangular for *R*. *palmatum*, and parted and lanceolate for *R*. *tanguticum* and *laciniatum*. Therefore, the range of AC/AB values was divided into three equal intervals. In other words, the theoretical maximum range of variation in AC/AB values was 0.2505 for each of the three morphological ranges, and the three variation ranges were 0.0970–0.3475, 0.3476–0.5980 and 0.5981–0.8485 (Tables 3, 4; Fig. 5A). Likewise, the CD/BE values ranged from 0.0873 to 2.0323 at the individual level, and the three variation ranges were 0.0873–0.7356, 0.7357–1.3839 and 1.3840–2.0323 (Tables 3, 4; Fig. 5B).

**Table 3:** Statistical parameters of the leaf blades of *R. palmatum* complex for 780 individuals of 44 populations.

Individuals	AC/AB	CD/BE	Individuals	AC/AB	CD/BE	Individuals	AC/AB	CD/BE	Individuals	AC/AB	CD/BE	Individuals	AC/AB	CD/BE
**Pop1-1**	0.6066	1.4000	Pop1-2	0.6667	1.7500	Pop1-3	0.5098	0.8462	Pop1-4	0.5946	1.2667	Pop1-5	0.6471	1.1333
Pop1-6	0.6829	1.6667	Pop1-7	0.8485	1.4286	Pop1-8	0.6364	1.6667	Pop1-9	0.5714	0.8125	Pop1-10	0.7059	1.4211
Pop1-11	0.5875	0.8235												
**Pop2-1**	0.6304	1.4464	Pop2-2	0.6264	1.5167	Pop2-3	0.6438	1.3082	Pop2-4	0.5564	0.9820	Pop2-5	0.6739	1.2987
**Pop3-1**	0.7167	1.6957	Pop3-2	0.6860	1.4865	Pop3-3	0.6667	1.5758	Pop3-4	0.6623	1.4167	Pop3-5	0.7333	1.5294
Pop3-6	0.7668	1.8235	Pop3-7	0.6291	1.4714	Pop3-8	0.5694	0.9931	Pop3-9	0.6936	1.6383	Pop3-10	0.6957	1.6190
Pop3-11	0.5693	1.0221	Pop3-12	0.6940	1.4068	Pop3-13	0.7143	1.4884	Pop3-14	0.6233	1.1961	Pop3-15	0.6375	1.2679
Pop3-16	0.6652	1.4563	Pop3-17	0.6118	1.1579									
**Pop4-1**	0.5467	1.0000	Pop4-2	0.5469	0.8140	Pop4-3	0.6604	1.4167	Pop4-4	0.7109	1.4286	Pop4-5	0.5000	0.8000
Pop4-6	0.5318	0.7778	Pop4-7	0.5621	0.9459	Pop4-8	0.5043	0.7377	Pop4-9	0.5697	0.9744	Pop4-10	0.5236	0.7200
Pop4-11	0.4946	0.7245	Pop4-12	0.7208	1.5185	Pop4-13	0.6588	1.2973	Pop4-14	0.6993	1.3704	Pop4-15	0.7089	1.3448
Pop4-16	0.6552	1.1778	Pop4-17	0.7037	1.1667	Pop4-18	0.6136	0.9756	Pop4-19	0.5054	0.7647	Pop4-20	0.6536	1.1333
**Pop5-1**	0.6389	1.3876	Pop5-2	0.6471	1.2981	Pop5-3	0.6408	1.1180	Pop5-4	0.5871	1.0185	Pop5-5	0.6538	1.1414
Pop5-6	0.5975	1.3250	Pop5-7	0.7113	1.7297	Pop5-8	0.7059	1.6237	Pop5-9	0.6053	1.1233	Pop5-10	0.5142	0.9550
Pop5-11	0.6275	1.6480	Pop5-12	0.6833	1.4716	Pop5-13	0.6163	1.2500	Pop5-14	0.7105	1.4127	Pop5-15	0.7075	1.4545
Pop5-16	0.4915	0.8563	Pop5-17	0.7387	1.3617	Pop5-18	0.5904	1.2222	Pop5-19	0.5556	0.9120			
**Pop6-1**	0.6628	1.2329	Pop6-2	0.6818	1.2000	Pop6-3	0.6893	1.4211	Pop6-4	0.7070	1.1750	Pop6-5	0.6757	1.3571
Pop6-6	0.7037	1.5556	Pop6-7	0.6990	1.4286	Pop6-8	0.7119	1.4091	Pop6-9	0.7297	1.7910	Pop6-10	0.7027	1.6333
Pop6-11	0.7364	1.5476	Pop6-12	0.6932	1.2958	Pop6-13	0.7257	1.5873	Pop6-14	0.6563	1.4545	Pop6-15	0.7333	1.8261
Pop6-16	0.6605	1.2527	Pop6-17	0.7262	1.6207	Pop6-18	0.7059	1.4583	Pop6-19	0.7095	1.6269			
**Pop7-1**	0.6723	1.1038	Pop7-2	0.5967	0.9697	Pop7-3	0.5800	0.8339	Pop7-4	0.5825	1.1379	Pop7-5	0.5868	0.9150
Pop7-6	0.5294	0.8894	Pop7-7	0.5842	0.9901	Pop7-8	0.5762	0.9466	Pop7-9	0.6703	0.9888	Pop7-10	0.6142	0.8957
Pop7-11	0.6318	0.7579	Pop7-12	0.5748	0.8496	Pop7-13	0.5729	0.8706	Pop7-14	0.5962	0.9381	Pop7-15	0.6406	1.0369
Pop7-16	0.6545	1.4300	Pop7-17	0.6105	0.9020	Pop7-18	0.5928	0.9836	Pop7-19	0.6722	1.0456			
**Pop8-1**	0.6047	1.3061	Pop8-2	0.6667	1.0000	Pop8-3	0.5814	0.8250	Pop8-4	0.4965	1.0441	Pop8-5	0.5859	1.0169
Pop8-6	0.5741	1.0000	Pop8-7	0.5882	1.1212	Pop8-8	0.5263	0.6250	Pop8-9	0.6119	1.1481	Pop8-10	0.5496	0.9286
Pop8-11	0.6167	1.2308	Pop8-12	0.5625	0.9375	Pop8-13	0.4918	0.7000	Pop8-14	0.6667	1.2000	Pop8-15	0.7292	1.6471
Pop8-16	0.6176	1.1875												
**Pop9-1**	0.5826	1.0862	Pop9-2	0.6033	1.0484	Pop9-3	0.5692	0.9870	Pop9-4	0.5182	0.8281	Pop9-5	0.6932	1.2826
Pop9-6	0.5306	1.3200	Pop9-7	0.5844	1.0417	Pop9-8	0.7209	1.0784	Pop9-9	0.5646	0.7848	Pop9-10	0.5250	0.8222
Pop9-11	0.5397	0.9091	Pop9-12	0.5865	1.0612	Pop9-13	0.5847	0.9355	Pop9-14	0.6333	0.9600	Pop9-15	0.5588	0.8462
Pop9-16	0.5763	0.9538	Pop9-17	0.5980	1.0000	Pop9-18	0.6458	1.2174	Pop9-19	0.5200	0.8780	Pop9-20	0.6854	0.9815
**Pop10-1**	0.7711	1.6552	Pop10-2	0.7103	1.6604	Pop10-3	0.7143	1.6667	Pop10-4	0.7088	1.7813	Pop10-5	0.7450	1.5472
Pop10-6	0.6468	1.3805	Pop10-7	0.6538	1.4000	Pop10-8	0.6774	1.2593						
**Pop11-1**	0.6327	1.2985	Pop11-2	0.5646	1.1127	Pop11-3	0.6131	1.2500	Pop11-4	0.6341	1.3393	Pop11-5	0.6347	1.1912
Pop11-6	0.6828	1.4098	Pop11-7	0.6618	1.2698	Pop11-8	0.6788	1.3714	Pop11-9	0.5679	1.1111	Pop11-10	0.6909	1.5000
Pop11-11	0.6429	1.2097	Pop11-12	0.6467	1.2647	Pop11-13	0.6245	1.1509	Pop11-14	0.5604	0.9474	Pop11-15	0.6824	1.4324
Pop11-16	0.5890	1.1500												
**Pop12-1**	0.6941	1.4468	Pop12-2	0.6894	1.3902	Pop12-3	0.7443	1.5114	Pop12-4	0.6651	1.3483	Pop12-5	0.6813	1.5333
Pop12-6	0.6692	0.9732	Pop12-7	0.6432	1.3263	Pop12-8	0.6316	1.2268	Pop12-9	0.6787	1.5682	Pop12-10	0.6923	1.5616
Pop12-11	0.7114	2.0000	Pop12-12	0.6576	1.4945	Pop12-13	0.7153	1.7115	Pop12-14	0.6899	1.5690	Pop12-15	0.6754	1.6321
Pop12-16	0.6725	1.5839												
**Pop13-1**	0.6087	0.9674	Pop13-2	0.6040	1.0047	Pop13-3	0.5952	0.9844	Pop13-4	0.5794	1.1769	Pop13-5	0.5399	1.1153
Pop13-6	0.5817	0.9572	Pop13-7	0.5854	1.1227	Pop13-8	0.7304	1.0870	Pop13-9	0.7544	1.1620	Pop13-10	0.6148	1.3157
Pop13-11	0.7655	1.4253	Pop13-12	0.5925	1.2140	Pop13-13	0.6243	1.1141	Pop13-14	0.7487	1.2049	Pop13-15	0.5671	1.0698
Pop13-16	0.6961	1.3349	Pop13-17	0.6507	1.1781	Pop13-18	0.6811	1.2939	Pop13-19	0.6241	1.1528	Pop13-20	0.6012	1.1804
**Pop14-1**	0.5376	0.8889	Pop14-2	0.6032	0.9751	Pop14-3	0.5542	1.0000	Pop14-4	0.6265	1.1995	Pop14-5	0.4412	0.6942
Pop14-6	0.4784	0.7385	Pop14-7	0.6499	1.5506	Pop14-8	0.6259	0.9111	Pop14-9	0.5270	0.6845	Pop14-10	0.5542	0.8025
Pop14-11	0.4995	0.7813	Pop14-12	0.5503	0.8108	Pop14-13	0.5104	0.8067	Pop14-14	0.5769	1.1125	Pop14-15	0.4748	0.6068
Pop14-16	0.6289	1.1799	Pop14-17	0.6488	1.4507	Pop14-18	0.4630	0.6828	Pop14-19	0.4716	0.5813	Pop14-20	0.6096	1.1923
**Pop15-1**	0.3889	0.5738	Pop15-2	0.4079	0.6122	Pop15-3	0.3976	0.5345	Pop15-4	0.3462	0.4444	Pop15-5	0.4000	0.5439
Pop15-6	0.4286	0.6230	Pop15-7	0.4646	0.7500	Pop15-8	0.5192	0.7458	Pop15-9	0.4783	0.6610	Pop15-10	0.5306	0.9643
Pop15-11	0.6279	1.2632	Pop15-12	0.5625	0.8261	Pop15-13	0.5682	0.9545	Pop15-14	0.5536	0.8889	Pop15-15	0.3763	0.5873
Pop15-16	0.4242	0.5738	Pop15-17	0.3457	0.5088	Pop15-18	0.3646	0.5417	Pop15-19	0.3882	0.5357	Pop15-20	0.4684	0.6122
**Pop16-1**	0.3814	0.5469	Pop16-2	0.4622	0.6004	Pop16-3	0.5352	0.8453	Pop16-4	0.3450	0.4778	Pop16-5	0.3396	0.4637
Pop16-6	0.5410	0.7904	Pop16-7	0.4394	0.6377	Pop16-8	0.3898	0.6591	Pop16-9	0.4234	0.6148	Pop16-10	0.5091	0.7913
Pop16-11	0.4547	0.8005	Pop16-12	0.5371	0.7980	Pop16-13	0.3548	0.4710	Pop16-14	0.4935	0.6659	Pop16-15	0.4082	0.5000
Pop16-16	0.4346	0.6242	Pop16-17	0.3551	0.4927	Pop16-18	0.4706	0.6589	Pop16-19	0.4908	0.7229	Pop16-20	0.4804	0.7086
**Pop17-1**	0.6013	1.0088	Pop17-2	0.6599	1.4526	Pop17-3	0.5571	0.8846	Pop17-4	0.5323	0.8407	Pop17-5	0.4321	0.5432
Pop17-6	0.3984	0.6220	Pop17-7	0.4239	0.5960	Pop17-8	0.4139	0.5435	Pop17-9	0.3809	0.4992	Pop17-10	0.5107	0.7802
Pop17-11	0.4014	0.4707	Pop17-12	0.5952	1.2143	Pop17-13	0.6920	1.3939	Pop17-14	0.6685	1.3990	Pop17-15	0.5998	0.9600
Pop17-16	0.5180	0.8438	Pop17-17	0.5388	0.9160	Pop17-18	0.4103	0.5338	Pop17-19	0.5594	0.8755			
**Pop18-1**	0.4451	0.6553	Pop18-2	0.4533	0.6225	Pop18-3	0.4522	0.6033	Pop18-4	0.5733	1.0313	Pop18-5	0.4849	0.7420
Pop18-6	0.6007	0.9953	Pop18-7	0.6605	1.4793	Pop18-8	0.4653	0.7536	Pop18-9	0.5636	0.9617	Pop18-10	0.5057	0.8798
Pop18-11	0.5314	0.7190	Pop18-12	0.4608	0.7713	Pop18-13	0.5277	0.8958	Pop18-14	0.5605	0.9245	Pop18-15	0.5799	0.9953
Pop18-16	0.4241	0.5697	Pop18-17	0.4553	0.7068	Pop18-18	0.4725	0.6842	Pop18-19	0.4961	0.8522	Pop18-20	0.5158	0.8676
**Pop19-1**	0.3931	0.6238	Pop19-2	0.4282	0.6410	Pop19-3	0.4416	0.6949	Pop19-4	0.2765	0.3478	Pop19-5	0.4691	0.7329
Pop19-6	0.3224	0.3974	Pop19-7	0.4386	0.6726	Pop19-8	0.3361	0.4138	Pop19-9	0.4398	0.6364	Pop19-10	0.3961	0.5458
Pop19-11	0.3822	0.4828	Pop19-12	0.3982	0.5711	Pop19-13	0.5327	0.8772	Pop19-14	0.5368	0.8964	Pop19-15	0.3868	0.5224
Pop19-16	0.3502	0.5772	Pop19-17	0.3242	0.4714	Pop19-18	0.3804	0.4747	Pop19-19	0.3156	0.4000	Pop19-20	0.4458	0.5610
**Pop20-1**	0.5274	0.7660	Pop20-2	0.6680	0.9510	Pop20-3	0.5018	0.9000	Pop20-4	0.6404	1.3136	Pop20-5	0.5781	0.9094
Pop20-6	0.5821	0.8150	Pop20-7	0.4874	0.8562	Pop20-8	0.6009	0.9831	Pop20-9	0.5730	0.8781	Pop20-10	0.5878	0.9916
Pop20-11	0.5977	0.9526	Pop20-12	0.5240	0.9569	Pop20-13	0.5538	1.1099	Pop20-14	0.5597	1.0125	Pop20-15	0.5434	0.9389
Pop20-16	0.5207	0.6946	Pop20-17	0.6458	0.9169	Pop20-18	0.6250	1.1521	Pop20-19	0.5195	0.8646	Pop20-20	0.5214	0.8158
**Pop21-1**	0.3627	0.4171	Pop21-2	0.4333	0.5912	Pop21-3	0.5067	0.8031	Pop21-4	0.3993	0.4712	Pop21-5	0.5165	0.7298
Pop21-6	0.3327	0.3852	Pop21-7	0.2717	0.2908	Pop21-8	0.3728	0.3997	Pop21-9	0.5222	0.7604	Pop21-10	0.3987	0.4831
Pop21-11	0.4467	0.5474	Pop21-12	0.5257	0.8476	Pop21-13	0.3716	0.4966	Pop21-14	0.3377	0.4860	Pop21-15	0.4439	0.7006
Pop21-16	0.2701	0.3345	Pop21-17	0.4137	0.5583	Pop21-18	0.4343	0.6318	Pop21-19	0.3656	0.3460	Pop21-20	0.3100	0.3291
**Pop22-1**	0.4858	0.7530	Pop22-2	0.4541	0.6988	Pop22-3	0.5405	0.7978	Pop22-4	0.7179	0.5276	Pop22-5	0.4969	0.7473
Pop22-6	0.6544	1.2754	Pop22-7	0.5504	0.8836	Pop22-8	0.5437	0.9792	Pop22-9	0.3971	0.6998	Pop22-10	0.5464	0.8679
Pop22-11	0.5781	0.9524	Pop22-12	0.3408	0.4553	Pop22-13	0.5079	0.7532	Pop22-14	0.3820	0.4676	Pop22-15	0.4050	0.7940
Pop22-16	0.6611	1.4822	Pop22-17	0.5345	0.8478	Pop22-18	0.6651	1.2556						
**Pop23-1**	0.4533	0.5238	Pop23-2	0.4286	0.4706	Pop23-3	0.5385	0.4118	Pop23-4	0.5556	0.7692	Pop23-5	0.4828	0.6250
Pop23-6	0.4737	0.6000	Pop23-7	0.4468	0.5185	Pop23-8	0.5000	0.6389	Pop23-9	0.5930	0.8571			
**Pop24-1**	0.4800	0.7000	Pop24-2	0.5500	0.8667	Pop24-3	0.5172	0.8667	Pop24-4	0.5758	0.9412	Pop24-5	0.5172	0.8065
Pop24-6	0.4583	0.5714	Pop24-7	0.5000	0.8182	Pop24-8	0.5143	0.7000	Pop24-9	0.4211	0.5833	Pop24-10	0.6154	1.3333
**Pop25-1**	0.6034	0.9737	Pop25-2	0.5470	0.9710	Pop25-3	0.5359	0.9082	Pop25-4	0.6183	1.1411	Pop25-5	0.6429	1.2733
Pop25-6	0.6362	1.0270	Pop25-7	0.6146	1.1781	Pop25-8	0.5751	0.9390	Pop25-9	0.5444	0.9618	Pop25-10	0.5941	0.9477
Pop25-11	0.5425	0.9648	Pop25-12	0.5685	0.8999	Pop25-13	0.6112	1.0819	Pop25-14	0.6085	0.9163	Pop25-15	0.6483	1.1639
Pop25-16	0.6030	0.8975	Pop25-17	0.5212	0.8370	Pop25-18	0.5240	0.8078	Pop25-19	0.4894	0.8484			
**Pop26-1**	0.5242	0.8889	Pop26-2	0.5275	0.9140	Pop26-3	0.4930	0.7236	Pop26-4	0.5657	0.8550	Pop26-5	0.5639	0.8941
Pop26-6	0.5217	0.8776	Pop26-7	0.5432	0.9474	Pop26-8	0.4606	0.6875	Pop26-9	0.4956	0.9630	Pop26-10	0.5062	0.6279
Pop26-11	0.6857	1.4286	Pop26-12	0.4400	0.6267	Pop26-13	0.4899	0.7841	Pop26-14	0.5088	0.7419			
**Pop27-1**	0.2865	0.3684	Pop27-2	0.3151	0.4182	Pop27-3	0.2963	0.3704	Pop27-4	0.3390	0.5119	Pop27-5	0.4052	0.6923
Pop27-6	0.3023	0.4545	Pop27-7	0.4685	0.8649	Pop27-8	0.4100	0.6970	Pop27-9	0.3579	0.6056	Pop27-10	0.3309	0.4468
Pop27-11	0.3778	0.5806	Pop27-12	0.3095	0.3967	Pop27-13	0.4348	0.6250	Pop27-14	0.3750	0.5229	Pop27-15	0.3529	0.5000
Pop27-16	0.4872	1.0204	Pop27-17	0.3614	0.5263	Pop27-18	0.4231	0.6957	Pop27-19	0.4217	0.6286			
**Pop28-1**	0.5085	0.7818	Pop28-2	0.4152	0.4962	Pop28-3	0.4529	0.5482	Pop28-4	0.4426	0.6111	Pop28-5	0.4369	0.5870
Pop28-6	0.4926	0.6281	Pop28-7	0.4350	0.5413	Pop28-8	0.3927	0.5164	Pop28-9	0.4797	0.5198	Pop28-10	0.4530	0.7202
Pop28-11	0.4779	0.7089	Pop28-12	0.4378	0.5719	Pop28-13	0.4324	0.4985	Pop28-14	0.5607	0.6633	Pop28-15	0.4797	0.5950
Pop28-16	0.4121	0.5170	Pop28-17	0.3829	0.5333	Pop28-18	0.4066	0.4808	Pop28-19	0.4398	0.4722	Pop28-20	0.5027	0.7289
**Pop29-1**	0.6080	1.0556	Pop29-2	0.5672	1.0000	Pop29-3	0.4667	0.7925	Pop29-4	0.5323	0.8824	Pop29-5	0.4105	0.6441
Pop29-6	0.5648	1.0000	Pop29-7	0.5526	1.0000	Pop29-8	0.5441	0.9189	Pop29-9	0.5172	1.2273	Pop29-10	0.5412	0.9302
Pop29-11	0.5042	0.8358	Pop29-12	0.5814	0.9756	Pop29-13	0.5362	0.8919	Pop29-14	0.4615	0.6400	Pop29-15	0.4754	0.7042
Pop29-16	0.5882	0.7627	Pop29-17	0.6296	0.8889	Pop29-18	0.5820	1.1935	Pop29-19	0.6027	1.2353	Pop29-20	0.5917	0.9180
**Pop30-1**	0.4736	0.6461	Pop30-2	0.4953	0.5630	Pop30-3	0.5019	0.7684	Pop30-4	0.5289	0.6128	Pop30-5	0.5310	0.6104
Pop30-6	0.5587	0.6894	Pop30-7	0.4628	0.5406	Pop30-8	0.4842	0.4797	Pop30-9	0.4905	0.7481	Pop30-10	0.5263	0.5781
Pop30-11	0.4663	0.6575	Pop30-12	0.4806	0.7748	Pop30-13	0.5060	0.7000	Pop30-14	0.4377	0.5438	Pop30-15	0.4417	0.5484
Pop30-16	0.5260	0.6603	Pop30-17	0.5076	0.7268	Pop30-18	0.5227	0.7441	Pop30-19	0.4603	0.7401	Pop30-20	0.4560	0.5548
**Pop31-1**	0.6858	1.4724	Pop31-2	0.6714	1.0251	Pop31-3	0.6376	1.0766	Pop31-4	0.5790	0.8497	Pop31-5	0.5480	1.0144
Pop31-6	0.5340	0.9273	Pop31-7	0.5276	0.7291	Pop31-8	0.5471	0.7806	Pop31-9	0.5772	0.8392	Pop31-10	0.6042	0.9004
Pop31-11	0.5505	1.0978	Pop31-12	0.5353	0.8589	Pop31-13	0.5393	0.7059	Pop31-14	0.5553	0.8408	Pop31-15	0.5422	0.8414
Pop31-16	0.5129	0.8275	Pop31-17	0.5151	0.6702	Pop31-18	0.5036	0.7692	Pop31-19	0.5655	0.8347	Pop31-20	0.5406	0.8559
**Pop32-1**	0.5685	0.6992	Pop32-2	0.4677	0.6766	Pop32-3	0.4312	0.5224	Pop32-4	0.4791	0.7425	Pop32-5	0.4782	0.8069
Pop32-6	0.5040	0.7541	Pop32-7	0.5091	0.7459	Pop32-8	0.5629	0.7058	Pop32-9	0.5919	0.8043	Pop32-10	0.5179	0.8696
Pop32-11	0.5260	0.7615	Pop32-12	0.5269	0.7686	Pop32-13	0.5217	0.8264	Pop32-14	0.5475	0.7893	Pop32-15	0.5335	0.8109
Pop32-16	0.5543	1.0032	Pop32-17	0.5635	0.8307	Pop32-18	0.5371	0.6708	Pop32-19	0.5915	0.7124	Pop32-20	0.5528	0.8114
**Pop33-1**	0.4213	0.6845	Pop33-2	0.5126	0.6711	Pop33-3	0.5140	0.9046	Pop33-4	0.4925	0.7237	Pop33-5	0.4541	0.7895
Pop33-6	0.4859	0.8937	Pop33-7	0.5146	0.9767	Pop33-8	0.5503	0.9861	Pop33-9	0.5857	1.0133	Pop33-10	0.4754	0.8053
Pop33-11	0.3975	0.6145	Pop33-12	0.3823	0.6718	Pop33-13	0.5518	0.6155	Pop33-14	0.5224	0.8220	Pop33-15	0.5044	0.8027
Pop33-16	0.5079	0.8700	Pop33-17	0.5133	0.8020	Pop33-18	0.5059	0.8874	Pop33-19	0.5102	0.9526	Pop33-20	0.4938	0.9208
**Pop34-1**	0.3224	0.4575	Pop34-2	0.3541	0.4555	Pop34-3	0.2976	0.3693	Pop34-4	0.3289	0.3958	Pop34-5	0.4080	0.5000
Pop34-6	0.4933	0.7784	Pop34-7	0.3778	0.5160	Pop34-8	0.4186	0.5991	Pop34-9	0.4019	0.2861	Pop34-10	0.3263	0.4689
Pop34-11	0.4739	0.5851	Pop34-12	0.4294	0.6271	Pop34-13	0.3350	0.3380	Pop34-14	0.3948	0.4766	Pop34-15	0.5677	0.7281
Pop34-16	0.4960	0.7429	Pop34-17	0.4611	0.5074	Pop34-18	0.3745	0.4040	Pop34-19	0.3433	0.4818	Pop34-20	0.4214	0.5109
**Pop35-1**	0.4304	0.7043	Pop35-2	0.4054	0.5997	Pop35-3	0.4617	0.6545	Pop35-4	0.4961	0.5738	Pop35-5	0.4173	0.5775
Pop35-6	0.3866	0.4751	Pop35-7	0.4110	0.5379	Pop35-8	0.3394	0.3996	Pop35-9	0.5554	0.7948	Pop35-10	0.3671	0.3959
Pop35-11	0.4090	0.5737	Pop35-12	0.3925	0.5962	Pop35-13	0.4108	0.4799	Pop35-14	0.4379	0.5813	Pop35-15	0.4047	0.4667
Pop35-16	0.4342	0.5974	Pop35-17	0.3860	0.5914	Pop35-18	0.3752	0.5096						
**Pop36-1**	0.4058	0.4948	Pop36-2	0.2980	0.4000	Pop36-3	0.3536	0.3552	Pop36-4	0.3597	0.4140	Pop36-5	0.3667	0.5525
Pop36-6	0.4370	0.6119	Pop36-7	0.3670	0.4855	Pop36-8	0.3244	0.4110	Pop36-9	0.4174	0.4751	Pop36-10	0.4238	0.5659
Pop36-11	0.3785	0.4365	Pop36-12	0.3500	0.5015	Pop36-13	0.3752	0.5512	Pop36-14	0.4841	0.6113	Pop36-15	0.4538	0.6655
Pop36-16	0.5120	0.7607	Pop36-17	0.3284	0.4199	Pop36-18	0.3950	0.6037						
**Pop37-1**	0.3659	0.4054	Pop37-2	0.3794	0.4474	Pop37-3	0.3114	0.3205	Pop37-4	0.3252	0.3570	Pop37-5	0.3089	0.2772
Pop37-6	0.2747	0.2671	Pop37-7	0.2663	0.3306	Pop37-8	0.3162	0.3078	Pop37-9	0.3802	0.2836	Pop37-10	0.3724	0.3187
Pop37-11	0.3399	0.3598	Pop37-12	0.4011	0.5238	Pop37-13	0.3527	0.3627	Pop37-14	0.3312	0.3626	Pop37-15	0.2325	0.3199
Pop37-16	0.4613	0.6552	Pop37-17	0.3013	0.3139	Pop37-18	0.3289	0.3837	Pop37-19	0.3298	0.3396			
**Pop38-1**	0.3765	0.6714	Pop38-2	0.3047	0.3360	Pop38-3	0.2963	0.3835	Pop38-4	0.4250	0.5994	Pop38-5	0.4065	0.5893
Pop38-6	0.2990	0.4850	Pop38-7	0.2799	0.4111	Pop38-8	0.3996	0.5658	Pop38-9	0.2578	0.3338	Pop38-10	0.2504	0.3081
Pop38-11	0.3389	0.5410	Pop38-12	0.3035	0.4327	Pop38-13	0.3560	0.4506	Pop38-14	0.3242	0.4143	Pop38-15	0.4096	0.3925
Pop38-16	0.3739	0.5045	Pop38-17	0.2773	0.5385	Pop38-18	0.4626	0.6849	Pop38-19	0.2775	0.3676	Pop38-20	0.2897	0.3753
**Pop39-1**	0.2500	0.3077	Pop39-2	0.3000	0.3636	Pop39-3	0.1373	0.1739	Pop39-4	0.1429	0.1316	Pop39-5	0.1923	0.0909
Pop39-6	0.2241	0.2727	Pop39-7	0.3784	0.5000	Pop39-8	0.1837	0.2051	Pop39-9	0.3256	0.3438	Pop39-10	0.1389	0.1724
Pop39-11	0.2000	0.1765	Pop39-12	0.1719	0.1724	Pop39-13	0.2381	0.3125						
**Pop40-1**	0.4783	0.3000	Pop40-2	0.2421	0.3026	Pop40-3	0.1972	0.2522	Pop40-4	0.4337	0.2982	Pop40-5	0.2622	0.2636
Pop40-6	0.4380	0.4643	Pop40-7	0.2816	0.3269	Pop40-8	0.2150	0.2414	Pop40-9	0.2389	0.2556	Pop40-10	0.2727	0.2605
Pop40-11	0.2092	0.2195	Pop40-12	0.2126	0.2057	Pop40-13	0.2482	0.2547	Pop40-14	0.2284	0.2358	Pop40-15	0.2212	0.3316
Pop40-16	0.2409	0.2074	Pop40-17	0.2723	0.3182	Pop40-18	0.2595	0.2381	Pop40-19	0.2464	0.1982			
**Pop41-1**	0.1834	0.2612	Pop41-2	0.1179	0.1441	Pop41-3	0.1234	0.1574	Pop41-4	0.2192	0.2443	Pop41-5	0.1729	0.2112
Pop41-6	0.2231	0.2261	Pop41-7	0.2077	0.2247	Pop41-8	0.1328	0.1323	Pop41-9	0.0970	0.0873	Pop41-10	0.2093	0.2345
Pop41-11	0.1268	0.1228	Pop41-12	0.1468	0.1358	Pop41-13	0.0992	0.0996	Pop41-14	0.1466	0.1620	Pop41-15	0.1772	0.1858
Pop41-16	0.2133	0.1837	Pop41-17	0.1734	0.1742	Pop41-18	0.1970	0.1994	Pop41-19	0.1721	0.2021	Pop41-20	0.1767	0.2068
**Pop42-1**	0.3087	0.4063	Pop42-2	0.2357	0.1892	Pop42-3	0.2774	0.3087	Pop42-4	0.3085	0.2975	Pop42-5	0.2783	0.2718
Pop42-6	0.2536	0.2363	Pop42-7	0.2733	0.2476	Pop42-8	0.2332	0.1938	Pop42-9	0.1442	0.1777	Pop42-10	0.1826	0.2082
Pop42-11	0.3216	0.3885	Pop42-12	0.2478	0.2706	Pop42-13	0.3148	0.3706	Pop42-14	0.2735	0.2828	Pop42-15	0.2345	0.2617
Pop42-16	0.2406	0.2553	Pop42-17	0.2171	0.2877	Pop42-18	0.2600	0.2844	Pop42-19	0.2612	0.2589	Pop42-20	0.2692	0.2478
**Pop43-1**	0.2698	0.2570	Pop43-2	0.2600	0.2505	Pop43-3	0.1994	0.2451	Pop43-4	0.2215	0.2012	Pop43-5	0.1885	0.2172
Pop43-6	0.1855	0.1909	Pop43-7	0.2064	0.2203	Pop43-8	0.2758	0.2970	Pop43-9	0.1958	0.2202	Pop43-10	0.1827	0.2792
Pop43-11	0.1744	0.2278	Pop43-12	0.1753	0.2287	Pop43-13	0.1865	0.1915	Pop43-14	0.1867	0.2062	Pop43-15	0.2254	0.2940
Pop43-16	0.2283	0.2510	Pop43-17	0.2491	0.2385	Pop43-18	0.2589	0.2482	Pop43-19	0.2494	0.2926	Pop43-20	0.2224	0.2578
**Pop44-1**	0.2321	0.2803	Pop44-2	0.2294	0.4205	Pop44-3	0.3051	0.4630	Pop44-4	0.2544	0.2373	Pop44-5	0.2687	0.2500
Pop44-6	0.3053	0.3478	Pop44-7	0.2778	0.3333	Pop44-8	0.2317	0.2154	Pop44-9	0.3365	0.3425	Pop44-10	0.2161	0.1915
Pop44-11	0.2606	0.2568	Pop44-12	0.2481	0.2100	Pop44-13	0.3364	0.2651	Pop44-14	0.2589	0.2898	Pop44-15	0.2444	0.2736
Pop44-16	0.3723	0.3731	Pop44-17	0.2679	0.1705	Pop44-18	0.2830	0.2791	Pop44-19	0.2115	0.2375			

**Table 4:** The variation ranges of AC/AB and CD/BE.

Values			AC/AB	CD/BE	Theoretical species
	Maximum		0.8485	2.0323	
	Minimum		0.0970	0.0873	
	Difference		0.7515	1.9450	
Individual level	Three-level interval		0.2505	0.6483	
		The first range	0.0970–0.3475	0.0873–0.7356	*R*. *officinale*
	Variation range	The second range	0.3476–0.5980	0.7357–1.3839	*R*. *palmatum*
		The third range	0.5981–0.8485	1.3840–2.0323	*R*. *tanguticum*, *R. laciniatum*
	Maximum		0.7271	1.6383	
	Minimum		0.1658	0.1798	
	Difference		0.5613	1.4585	
Population level	Three-level interval		0.1871	0.4862	
		The first range	0.1658–0.3529	0.1798–0.6660	*R*. *officinale*
	Variation range	The second range	0.3530–0.5400	0.6661–1.1522	*R*. *palmatum*
		The third range	0.5401–0.7271	1.1523–1.6383	*R*. *tanguticum*, *R. laciniatum*

The AC/AB values ranged from 0.1658 to 0.7271 at the population level in the *R. palmatum* complex (Table 4), and the difference between the maximum and minimum values was 0.5613. The difference of AC/AB values was divided into three equal intervals. In other words, the theoretical maximum variation range of AC/AB values was 0.1871 for each of the three morphological ranges, and the three variation ranges were 0.1658–0.3529, 0.3530–0.5400 and 0.5401–0.7271. These three ranges should theoretically correspond to the depth of the blade division of *R. officinale*, *R. palmatum* and *R. tanguticum*, respectively. Similarly, the CD/BE values ranged from 0.1798 to 1.6383 at the population level (Table 4), and the difference between the maximum and minimum values was 1.4585. The difference of the CD/BE value was also divided into three equal intervals. In other words, the theoretical maximum variation range of CD/BE values was 0.4862 for each of the three morphological ranges, and the three variation ranges were 0.1798–0.6660, 0.6661–1.1522 and 1.1523–1.6383. Based on the mean AC/AB and CD/BE values at the population level, the minimum variation of AC/AB and CD/BE values occurred in populations Pop37-Pop44, and these populations possessed deeper clefts and narrower lobes, corresponding to *R. tanguticum* (including its variety) and *R. laciniatum*. For the maximum and the moderate variation ranges, Pop1-Pop6 and Pop10-Pop13 were included in the maximum variation range that corresponded with *R. officinale* both for the AC/AB value and for the CD/BE value, but Pop7 and Pop8 were exceptions; seventeen populations, i.e., Pop 15-Pop19, Pop21-Pop24, Pop26-Pop28, Pop30 and Pop33-Pop36, were within the moderate variation range, which corresponded to *R. palmatum*. However, six populations, Pop9, Pop14, Pop20, Pop25, Pop29 and Pop31, were within the moderate variation range based on the AC/AB value but belonged to the maximum variation range for the CD/BE value. Pop7 and Pop8 differed from the above populations, residing within the maximum variation range of the AC/AB value; however, these two populations were within the moderate variation range and the maximum variation range of CD/BE values, respectively. These results indicate that these eight populations, Pop7, Pop8, Pop9, Pop14, Pop20, Pop25, Pop29 and Pop31, are the transitional forms between *R. officinale* and *R. palmatum.*

The two indices of the leaf blade cleft also differed greatly among the different individuals within populations, and the differences between populations were also not identical (Table 5; Fig. 3). The difference in the AC/AB values varied from 0.0959 to 0.3771, among which the differences for nine populations were larger than the trisection of the difference between the maximum and minimum of AC/AB values (0.2505). The population with the smallest difference was Pop43, followed by Pop41, and that with the largest difference was Pop22, followed by Pop12. The difference in the CD/BE values varied from 0.1061 to 1.0269, among which the differences for 18 populations were larger than the trisection of the difference between the maximum and minimum of the CD/BE values (0.6483). The population with the smallest difference was Pop6, followed by Pop43, and that with the largest difference was Pop22, followed by Pop16. The difference had no correlation with the population size.

**Table 5:** AC/AB and CD/BE value at population level.

Population	AC/AB				CD/BE			
	Mean	Maximum	Minimum	Difference	Mean	Maximum	Minimum	Difference
Pop1	0.6416	0.8485	0.5098	0.3387	1.2923	1.7500	0.8235	0.9265
Pop2	0.6262	0.6739	0.5564	0.1175	1.3104	1.5167	0.9820	0.5347
Pop3	0.6668	0.7668	0.5693	0.1975	1.4262	1.8235	0.9931	0.8304
Pop4	0.6035	0.7208	0.4946	0.2262	1.0544	1.5185	0.7200	0.7985
Pop5	0.6238	0.7387	0.4915	0.2472	1.2794	1.7297	0.8563	0.8734
Pop6	0.7031	0.7522	0.6563	0.0959	1.4950	2.0270	1.1750	0.852
Pop7	0.6073	0.6723	0.5294	0.1429	0.9729	1.4300	0.7579	0.6721
Pop8	0.5919	0.7292	0.4918	0.2374	1.5074	1.6471	0.6250	1.0221
Pop9	0.5910	0.7209	0.5182	0.2027	1.0011	1.3200	0.7848	0.5352
Pop10	0.7271	0.8333	0.6468	0.1865	1.6383	2.0323	1.2593	0.773
Pop11	0.6317	0.6909	0.5604	0.1305	1.2506	1.5000	0.9474	0.5526
Pop12	0.6820	0.7443	0.6316	0.1127	1.4923	2.0000	0.9732	1.0268
Pop13	0.6373	0.7655	0.5399	0.2256	1.1531	1.4253	0.9572	0.4681
Pop14	0.5516	0.6499	0.4412	0.2087	0.9325	1.5506	0.5813	0.9693
Pop15	0.4521	0.6279	0.3457	0.2822	0.6872	1.2632	0.4444	0.8188
Pop16	0.4423	0.5410	0.3396	0.3396	0.6435	0.8453	0.4637	0.3816
Pop17	0.5207	0.6920	0.3809	0.3111	0.8620	1.4526	0.4707	0.9819
Pop18	0.5114	0.6605	0.4241	0.2364	0.8355	1.4793	0.5697	0.9096
Pop19	0.3997	0.5368	0.2765	0.2603	0.5770	0.8964	0.3478	0.5486
Pop20	0.5679	0.6680	0.4874	0.1806	0.9389	1.3136	0.6946	0.619
Pop21	0.4018	0.5257	0.2701	0.2556	0.5305	0.8476	0.2908	0.5568
Pop22	0.5257	0.7179	0.3408	0.3771	0.8466	1.4822	0.4553	1.0269
Pop23	0.4969	0.5930	0.4286	0.1644	0.6017	0.8571	0.4118	0.4453
Pop24	0.5149	0.6154	0.4211	0.1943	0.8187	1.3333	0.5714	0.7619
Pop25	0.5804	0.6483	0.4894	0.1589	0.9862	1.2733	0.8078	0.4655
Pop26	0.5233	0.6857	0.4400	0.2457	0.8543	1.4286	0.6267	0.8019
Pop27	0.3713	0.4872	0.2865	0.2007	0.5751	1.0204	0.3684	0.652
Pop28	0.4521	0.5607	0.3829	0.1778	0.5860	0.7818	0.4722	0.3096
Pop29	0.5429	0.6296	0.4105	0.2191	0.9248	1.2353	0.6400	0.5953
Pop30	0.4929	0.5587	0.4377	0.121	0.6444	0.7748	0.4797	0.2951
Pop31	0.5636	0.6858	0.5036	0.1822	0.8959	1.4724	0.6702	0.8022
Pop32	0.5283	0.5919	0.4312	0.1607	0.7656	1.0032	0.5224	0.4808
Pop33	0.4948	0.5857	0.3823	0.2034	0.8204	1.0133	0.6145	0.3988
Pop34	0.4013	0.5677	0.2976	0.2701	0.5114	0.7784	0.2861	0.4923
Pop35	0.4178	0.5554	0.3394	0.216	0.5616	0.7948	0.3959	0.3989
Pop36	0.3906	0.5120	0.2980	0.214	0.5176	0.7607	0.3552	0.4055
Pop37	0.3357	0.4613	0.2325	0.2288	0.3651	0.6552	0.2671	0.3881
Pop38	0.3354	0.4626	0.2504	0.2122	0.4693	0.6849	0.3081	0.3768
Pop39	0.2218	0.3784	0.1373	0.2411	0.2479	0.5000	0.0909	0.4091
Pop40	0.2736	0.4783	0.1972	0.2811	0.2723	0.4643	0.1982	0.2661
Pop41	0.1658	0.2231	0.0970	0.1261	0.1798	0.2612	0.0873	0.1739
Pop42	0.2568	0.3216	0.1442	0.1774	0.2723	0.4063	0.1777	0.2286
Pop43	0.2171	0.2758	0.1744	0.1014	0.2407	0.2970	0.1909	0.1061
Pop44	0.2706	0.3723	0.2115	0.1608	0.2861	0.4630	0.1705	0.2925

### Relationships between leaf variations and elevation, longitude and latitude

Correlation analysis revealed that the AC/AB and DC/BE values were both significantly correlated with latitudes and altitudes (Table 2, *P*<0.01and *P*<0.05, respectively). With higher latitudes and/or altitudes, the blade divisions became deeper, and the blade lobes became narrower, especially on a single mountain, such as the four populations on the north slope of Mt. Taibai, Shaanxi province. The indices of leaf division showed no significant correlation with the longitudes or with the different longitudes at approximately the same latitudes.

In general, individuals from populations in the southeastern area of the distribution of the species complex tended to have shallower lateral clefts of leaf blades and wider central lobes than those from the northwestern area. The differences among all of the populations are gradational rather than distinct.

## Discussion

Traditional classification and species delimitation are mainly based on herbarium specimens, and this strategy introduces many limitations. For example, herbarium specimens are often partial but not entire plants; furthermore, very few specimens are collected from the same location, rendering it difficult to reflect the within-population variation. Coupled with the impact of the concept of type species, some scholars believe that a specimen can represent all the characteristics of a given species. Many new species are established based on the differences between the specimens in hand and holotype according to one or two morphological traits. Individuals that are distinct from the holotype cannot be established as new taxa [2].

In the present study, the morphological traits of leaf blades for discriminating the species in *R*. *palmatum* complex were analyzed at the population level. Unfortunately, species within the species complex cannot be discriminated by the degree of leaf blade dissection and the shape of the lobes. As shown in Figure 3, the depth of the lateral cleft (represented by AC/AB) and the width of the central lobe (represented by DC/BE) are continuous. The present study indicates that the two indices (AC/AB and DC/BE) are continuous among different species of the complex, and even the within-population variation could be larger than the interpecific variation. Our findings are consistent with those of previous morphological studies. Ge and Hong [5], [7] studied morphological characteristics such as leaf shape, tooth number and size of leaf margins of *Adenophora potaninii* complex, and no discontinuities were found among them. Based on their findings, they recognized only one species and two subspecies within the species complex. Yang et al. [27] investigated the morphological variation pattern of *Medicago sativa* complex, and they showed that the morphology of stems and leaves was too similar to be the key characteristics for the complex.

Leaves are photosynthetic organs. To absorb sufficient light energy, leaves must be as wide as possible. Meanwhile, to facilitate gas exchange (CO_2_, O_2_ and H_2_O), leaves tend to be flat and thin. Therefore, leaves become the organ with the largest contact area with the environment, and they are thus heavily influenced by the environment. Many quantitative characteristics in plants, including size, weight and number, are controlled by polygenes [28], and the variation of these characteristics is continuous within and/or among populations. Therefore, these characteristics are less meaningful for species delimitation. Although such quantitative characteristics are greatly influenced by the environment, their intrinsic genetic variety may not be high. Thus, they are not good taxonomic characteristics [2]. The traditional identification of *R*. *palmatum* complex was indeed based on unreliable morphological characteristics. Morphological characteristics based on the degree of leaf blade dissection and the lobe shape are continuous among and within the studied populations. Yang and Zhang published a new species, *R. qinlingense*, which was later treated as a synonym of *R. palmatum* [29]. According to our analysis, *R. qinlingense* actually represents the intermediate type of *R. officinale* and *R. palmatum*. Wu et al. [29] also suggested that *R. officinale* and *R. palmatum* as recorded in *Flora Tsinlingensis* [30] should actually be considered as a single species. Our field survey showed that different leaf blades described as *R. officinale* and *R. palmatum* can be found within a single population, such as Pop14 (Xiabansi, Mt. Taibai, the major peak of Mts. Qinling). This phenomenon can also be found in *R. palmatum* and *R. tanguticum*, such as Pop38 (Jimai Village, Dari County, Qinghai Province). As described in Flora of China [20], *R. laciniatum* differs from *R. tanguticum* only in the shape of the lobelets. *R. laciniatum* is distributed in the northern Sichuan Province; unfortunately, we failed to find a specimen of *R. laciniatum* in any of the herbaria in China. According to the scatter diagram or the histogram, it is clear that *R. laciniatum* is not a separate entity but an extreme form of *R. tanguticum*. Likewise, *R. tanguticum* var. *liupanshanense* also represents an extreme form of *R. tanguticum* var. *tanguticum.* Similar findings also exist for other taxa, e.g., Gallego et al. [31]. Our analysis did not support *R. officinale*, *R. tanguticum* or *R. laciniatum* as independent species. It appears more reasonable to recognize these species as synonyms of *R. palmatum*.

AC/AB and DC/BE values were found to be significantly correlated with each other, indicating that the degree of leaf blade dissection and the shape of the lobes were not meaningful for identifying the species in *R*. *palmatum* complex. Moreover, AC/AB and DC/BE were both significantly correlated with latitudes, altitudes and longitudes (Table 2). Continuous variation in morphological characteristics among species in *R*. *palmatum* complex may be caused by geographical and ecological factors (e.g., altitude, latitude, longitude). With the latitude rising, the growth environment of *R*. *palmatum* complex becomes increasingly dry. While the leaves of *R*. *palmatum* complex are relatively large, so in order to reduce transpiration area, the leaf lobes of *R*. *palmatum* complex become deeper and narrower. The change of the altitude is similar with that of the latitude.To date, many reports have dealt with the relationships among *R. officinale*, *R. tanguticum*, and *R. palmatum* at the molecular level as described in the introduction. It should be noted that these studies [22]–[24] have involved only a limited number of samples for each species. It is clear that the morphological analysis must be complemented by an analysis of molecular characteristics, such as ITS (internal transcribed spacer) sequences or single (or low) copy nuclear genes, for better resolution within Sect. *Palmata* and to examine the interspecific relationships proposed in this study. A molecular study is currently underway to gain further insight into this issue.

As for *R*. *laciniatum*, its difference from *R*. *tanguticum* lies only in the shape of the lobelets, which are linear and lanceolate for *R*. *laciniatum* and *R*. *tanguticum*, respectively. Although the terminal lobes were not analyzed in the present study, leaf lobe types, which varied from no secondary lobes to trilobate lobes, differ greatly among different populations, among different individuals within the same population, and even among different blades of the same individual. Whether secondary lobes or trilobate lobes, their shape also varied from triangular to narrowly lanceolate.

In summary, we suggest that *R*. *palmatum* complex be considered as a single species. *Rheum officinale*, *R*. *tanguticum* (including var. *liupanshanense*) and *R*. *laciniatum* are synonyms of *R*. *palmatum*. However, our hypothesis has yet to be confirmed by further studies, possibly using various molecular markers.
